# Medicated Bougies for Gonorrhœa

**Published:** 1868

**Authors:** 


### MEDICATED BOUGIES FOR GONORRHŒA.

Dr. Bartholow (*Cincinnati Repertory*) employs with great benefit in gonorrhœa, after the subsidence of the first stage, and in gleet, bougies of cocoa butter variously medicated. Tannic acid, acetate of lead, morphia, and other remedies may be thus applied directly to the disordered surface. In summer it is necessary to add a little wax to the butter. The bougie should be about four inches long, and large enough to fill the urethra without distending it. If it tend to slip out, a strip of adhesive plaster can be applied. The best time for its use is at night.—*Pacific Medical Journal.*

